# Disrupting of IGF2BP3-stabilized CLDN11 mRNA by TNF-α increases intestinal permeability in obesity-related severe acute pancreatitis

**DOI:** 10.1186/s10020-025-01078-9

**Published:** 2025-01-24

**Authors:** Lihui Lin, Yansong Lin, Xianwen Guo, Ruoyi Zhang, Xin Ling, Zewen Zhang, Rong Lin, Zhen Ding

**Affiliations:** 1 Division of Gastroenterology, The First Affiliated Hospital, Sun Yat-Sen University, Guangzhou, P. R. China; 2 Department of Pathology, Sun Yat-Sen University Cancer Center, Guangzhou, P. R. China; 3 Department of Gastroenterology, Union Hospital, Tongji Medical College, Huazhong University of Science and Technology, 1277 Jiefang Avenue, Wuhan, P. R. China

**Keywords:** Obesity, Severe acute pancreatitis, Intestinal permeability, CLDN11, IGF2BP3, TNF-α

## Abstract

**Background:**

Obesity is a significant risk factor for severe acute pancreatitis (SAP) and is typically associated with increased intestinal permeability. Understanding the role of specific molecules can help reduce the risk of developing SAP. Claudin 11 (CLDN11), a member of the Claudin family, regulates the permeability of various internal barriers. However, the role and mechanism of CLDN11 in the intestinal permeability of obesity-related SAP remain unclear.

**Methods:**

We evaluated intestinal permeability and the expression of CLDN11 in experimental obesity-related SAP. A recombinant adeno-associated virus carrying CLDN11 was used to treat experimental obesity-related SAP. The interaction between CLDN11 mRNA and insulin-like growth factor 2 mRNA-binding protein 3 (IGF2BP3) protein was predicted through bioinformatics analysis and validated by RNA immunoprecipitation and RNA pull-down assay. Additionally, tumor necrosis factor-α (TNF-α) treatment in Caco-2 cells was conducted, and the IGF2BP3/CLDN11 axis was detected. Moreover, we conducted anti-TNFα therapy and evaluated intestinal permeability and pancreatic inflammation in experimental obesity-related SAP.

**Results:**

Downregulation of CLDN11 was observed in the intestinal epithelial cells of experimental obesity-related SAP. When the expression of CLDN11 in intestinal epithelial cells of experimental obesity-related SAP was increased exogenously, intestinal epithelial permeability and pancreatic inflammation were relieved. Overexpression of CLDN11 reduced the paracellular permeability of Caco-2 monolayer cells, while knockdown of CLDN11 increased it. IGF2BP3 bound to and regulated the stability of CLDN11 mRNA. TNF-α treatment downregulated IGF2BP3 and CLDN11 in vitro. Anti-TNFα therapy reduced intestinal permeability, alleviated pancreatitis, and improved the expression of IGF2BP3 and CLDN11 in intestinal epithelial cells in experimental obesity-related SAP.

**Conclusion:**

CLDN11 regulates intestinal permeability in obesity-related SAP. Mechanistically, an increase in TNF-α impaired the stability of IGF2BP3-dependent CLDN11 mRNA in obesity-related SAP.

**Supplementary Information:**

The online version contains supplementary material available at 10.1186/s10020-025-01078-9.

## Introduction

Acute pancreatitis (AP) is an inflammatory injury of the pancreas characterized by edema, bleeding and necrosis (Vege et al. 2018). According to the Atlanta classification system, severe acute pancreatitis (SAP) refers to organ failure that persists for > 48 hours (h) or infectious pancreatic necrosis. Compared to mild AP, which has a mortality rate of < 1% (Uhl et al. 2002), SAP has a much higher mortality rate: sterile SAP, 10%; infectious SAP, 25% (Dervenis et al. 1999). Patients with SAP typically require hospitalization for > 2 weeks and often need to be admitted to intensive care unit (Swaroop et al. 2004).

Obesity increased the risk of local and systemic complications in AP, exacerbating its severity, leading to poor prognosis, and potentially resulting in death (Papachristou et al. 2006; Shin et al. 2011; Sempere et al. 2008; Abu et al. 2008). Further researches indicated that obesity can mediate SAP progression by downregulating intestinal tight junctions or altering the gut microbiome, thereby disrupting intestinal barriers and facilitating bacterial or endotoxin translocation (Ye et al. 2019; Huang et al. 2023). However, the specific tight junctions involved in obesity-related SAP and the underlying mechanisms remain unclear.

Claudin 11 (CLDN11) is a 4-fold transmembrane protein. It has been reported that CLDN11 maintained the integrity of a Caco-2 monolayer model (Li et al. 2021). Additionally, CLDN11 regulated bone homeostasis (Lindsey et al. 2019; Baek et al. 2018), mediated the normal function of oligodendrocytes (Maheras et al. 2018), and participated in spermatogenic disorders (Haverfield et al. 2013). It has been reported that the expression of CLDN11 can be regulated by transcription factors (Li et al. 2019), methylated promoters (Abe et al. 2016), and microRNA targeting (Yamada et al. 2019). However, research on the specific roles and regulatory mechanisms of CLDN11 in obesity-related SAP remains limited.

Insulin-like growth factor 2 mRNA-binding protein 3 (IGF2BP3) is an RNA-binding protein that controls the transcription and stability of the target mRNA (Lederer et al. 2014). Since the first identification of IGF2BP3, numerous studies have reported its abnormal expression in human cancers (Findeis-Hosey et al. 2011); however, recent studies have confirmed its involvement in regulating inflammation. Lu et al. (Lu et al. 2023) reported that IGF2BP3 was upregulated in the synovium of patients with osteoarthritis, promoting macrophage M1 polarization and inflammation. In addition, IGF2BP3 activated nuclear factor kappa-B (NF-κB) signal transduction and damaged retinal pigment epithelial cells (Tian et al. 2023).

In this study, we aimed to clarify the role of CLDN11 in the intestinal epithelial cells of obesity-related SAP and investigate the regulatory mechanism of IGF2BP3 on CLDN11 under inflammatory conditions.

## Methods and materials

### Obesity-related SAP mouse model establishment

All animal experiments were approved by the Ethical Committee of the Laboratory Animal Center of Sun Yat-sen University (2023000734). Four-week-old male C57BL/6 mice were purchased from Zhuhai BesTest Bio-Tech Co. Ltd (Zhuhai, China). The mice were housed in a standard laboratory environment with a temperature of 22 ± 2 ℃, humidity of ± 10%, and a light/dark cycle of 12 h. Mice were fed a regular diet (10 kcal% fat) or a high-fat diet (60 kcal% fat, Ready Biotechnology, Shenzhen, China) with sufficient water for 16 weeks. To further induction of SAP in obese mice, their body weight needed to be at least 30% heavier than that of mice fed a regular diet. SAP was induced using caerulein (CAE) (MedChemExpress, California, USA) and lipopolysaccharide (LPS) (Biosharp, Hebei, China). The CAE and LPS were dissolved in sterile saline and kept in an icebox. Mice received intraperitoneal injections of 50 µg/kg/h CAE, administered a total of 7 times. Following the last CAE injection, 10 mg/kg LPS was immediately administered. The control group was administered saline based on body weight. Blood, pancreatic, and colon tissues were collected 24 h after the induction of pancreatitis.

### Measurement of intestinal permeability in vivo

A fully functional intestine acts as a barrier against bacterial metabolites such as *D*-lactate and endotoxins, preventing them from entering the bloodstream. The presence of these metabolites in the bloodstream is indicative of increased intestinal permeability. Therefore, *D*-lactate and endotoxin were used to detect intestinal permeability according to the manufacturer’s protocols (AAT Bioquest, California, USA).

### Bacterial translocation

Fluorescence in situ hybridization was used to determine bacterial translocation. Pancreas and colon tissue sections were dewaxed (56 ℃ 30 min; xylene three times, 5 min each), dehydrated (70%, 80%, 90%, and 100% ethanol, 5 min each), and air dried. Pre-hybridization solution was applied to the tissue sections and incubated at 37 ℃ for 30 min. The bacterial universal probe (EUB338: 5’-Cy3-GCTGCCTCCCGTAGGAGT-3’ [Mei et al. 2022]) was mixed with a hybridization solution in a 1:39 ratio to prepare the hybridization reaction solution. Tissue slices were then added the hybridization reaction solution, denatured at 73 ℃ for 8 min, and then incubated at 37 ℃ overnight in a hybrid oven (Advanced Cell Diagnostics, California, USA). A laser confocal microscope (Olympus, Tokyo, Japan) was used to capture the images.

### Quantitative real-time polymerase chain reaction (qPCR)

Total RNA was extracted from tissues and cultured cells using RNA extraction reagent (Accurate, Changsha, China). RNA concentration was measured using a Nanodrop spectrophotometer (Thermo Scientific, Massachusetts, USA). RNA was reverse transcribed into cDNA according to the manufacturer’s protocol. Quantitative PCR was performed to examine gene expression using a LightCycler 480 fluorescence qPCR machine (Thermo Scientific). β-actin was used as internal references; primer sequences of target genes and β-actin are listed in Supplemental Table S1.

### Western blotting (WB)

Total protein was extracted from tissues and cultured cells using a cell lysis buffer (Beyotime, Shanghai, China) and a protease inhibitor cocktail (Thermo Scientific). The protein concentration was measured using a bicinchoninic acid protein assay (Thermo Scientific). Proteins were then electrophoresed on 12.5% sodium dodecyl sulfate-polyacrylamide gel electrophoresis and transferred onto polyvinylidene fluoride membranes. The membranes were blocked with 5% skim milk and incubated overnight with primary antibodies at 4 ℃. Antibodies are listed in Supplemental Table S2. Subsequently, the membranes were washed with Tris-buffered saline containing Tween 20 and incubated with horseradish peroxidase-conjugated anti-rabbit/mouse IgG antibodies for 1 h. Protein bands were visualized using a chemiluminescence imaging system (Thermo Scientific). Protein levels were normalized to β-actin as an internal reference.

### Immunohistochemistry (IHC) and immunofluorescence (IF)

For IHC staining, colon tissue sections were dewaxed (56 ℃ for 30 min; xylene three times, 5 min each), hydrated (100%, 95%, and 75% ethanol, 5 min each), and washed with double distilled water 3 times. Based on the primary antibodies’ instructions, appropriate antigen retrieval solutions (Tris-ethylenediaminetetraacetic acid or citrate sodium) were used. Endogenous peroxidase activity was blocked. The primary antibodies were incubated overnight at 4 ℃, and anti-species secondary antibodies were incubated for 30 min. Finally, the slices were stained with a hematoxylin solution.

For double IF staining, sections were incubated with primary antibodies overnight at 4 ℃, followed by specific fluorescence-conjugated secondary antibodies. Antibodies are listed in Supplemental Table S2.

### Cell culture and co-culture cell system

The human colon adenocarcinoma cell line Caco-2 and human pancreatic carcinoma cell line PANC-1 were purchased from the American Type Culture Collection (ATCC, Virginia, USA). The Caco-2 and PANC-1 cells were cultured in Dulbecco’s modified Eagle’s medium with 10% fetal calf serum and placed in a 37 ℃, 5% CO_2_ incubator (Thermo Scientific).

A Caco-2 and PANC-1 co-culture system was established using a 0.4 μm polyester transwell chamber and a matching 6-well plate. The PANC-1 cells with 10 nM CAE and 0.5 mM palmitic acid (PA) (Sigma-Aldrich, Missouri, USA) were incubated in the co-culture system with Caco-2 cells. The control group was incubated with equal amounts of saline and fatty acid-free bovine serum albumin.

### Stable Caco-2 cell lines establishment

To construct *CLDN11* and *IGF2BP3* overexpression (OE) plasmids, the coding sequences of human *CLDN11* and *IGF2BP3* were amplified and cloned into the pLVX-puro lentivirus vector (#118692, Addgene, Massachusetts, USA). An empty vector served as a control. To construct *CLDN11*, *IGF2BP3*, *methyltransferase like protein 3 (METTL3)*, and *methyltransferase like protein 14 (METTL14)* knockdown plasmids, short hairpin RNAs were cloned into a pLKD-puro lentivirus vector (#8453, Addgene). A control short hairpin RNA was also used. The short hairpin RNA sequences are listed in Supplemental Table S3. According to the manufacturer’s protocols, the plasmids were transfected into HEK293T cells using Lipofectamine™ 3000 (Invitrogen, California, USA). After 48 h of transfection, the lentivirus supernatant was collected, filtered using 0.22 μm filters (Millipore, Massachusetts, USA), and used to transfect Caco-2 cells. The transfected cells were selected with 10 µg/mL puromycin (Solarbio, Beijing, China) for 1 week.

### Transepithelial resistance (TER) and fluorescein isothiocyanate-dextran 4 kDa (FD4) permeability assessment

TER was performed to assess paracellular permeability in vitro. Caco-2 cells were seeded in the upper chamber of a 6-well Transwell chamber at a density of 5 × 10^4^ cells per well. Millicell ERS-2 (Millipore) was used to perform the TER assessment. A Caco-2 cell monolayer model with TER ≥ 200Ω × cm^2^ was considered successfully established.

FD4 (Sigma-Aldrich) was used to assess the permeability of the Caco-2 cell monolayer. When the Caco-2 cell monolayer model matured, 1 mL Opti-MEM (Gibco, California, USA) containing FD4 (2 mg/mL) was added to the upper chamber and 2 mL Opti-MEM to the lower chamber. The fluorescence intensity in the lower chamber was measured using a spectrophotometer (PerkinElmer).

### Adeno-associated virus9 (AAV9) construction and injection

The exogenous gene *CLDN11* was cloned into an AAV9 vector. The recombinant plasmid carrying the *CLDN11* gene, along with the helper plasmids Ad Helper Vector and pAAV-rep/capVector, were co-transfected into HEK293T packaging cells. After 72 h of transfection, the supernatant and cell pellets were collected separately. The virus in the culture supernatant was precipitated using PEG8000, and the virus in the cell pellet was collected after cell lysis. The virus obtained from both the cell pellet and the supernatant was pooled together. Virus purification was performed using iodixanol density gradient centrifugation. After purification, the collected viral solution was further concentrated by ultrafiltration. The viral titer was determined by qPCR, the virus specificity was confirmed by PCR, and the viral purity was assessed using silver staining. The successfully constructed AAV was properly stored in a -80 °C freezer until use. During the establishment of the obesity model and the 2 to 3 weeks prior to the induction of pancreatitis, 9 × 10^11^ physical particles of AAV9 was administered via tail vein injection into the mice. After injection, the mice were housed in standard animal experimental facilities.

### Detection of biochemical indicators and pro-inflammatory cytokines

According to the manufacturer’s protocols (Jiangsu Meimian Industrial Co., Ltd), mice bloods were processed into serum after centrifugation. Serum was then used to detect biochemical indicators (AMY [amylase], TG [triglyceride], and TC [total cholesterol]) and pro-inflammatory cytokines (TNF-α, interleukin-6 [IL-6], interleukin-1β [IL-1β], and interferon-γ [IFN-γ]). Pancreatic samples were used to examine myeloperoxidase (MPO) activities, which were normalized to protein content.

### Histopathological assessment

Pancreas and colon tissues fixed with 4% paraformaldehyde were embedded into paraffin blocks and sectioned into 4 μm thick sections for hematoxylin and eosin staining. Interstitial edema, acinar necrosis, bleeding, fat necrosis, and inflammatory cell infiltration in the pancreatic tissue were scored based on the scoring system for experimental acute pancreatitis (Schmidt et al. 1992).

### Prediction of protein-CLDN11 mRNA pairs

The protein-CLDN11 mRNA pairs were predicted using catRAPID (http://service.tartaglialab.com/page/catrapid_group). The model structure of IGF2BP3 protein was downloaded from the Uniprot database (https://www.uniprot.org), and the mRNA sequence of CLDN11 was acquired from the PubMed database (http://pubmed.nubi.nlm.nih.gov). We employed the FASTA sequence of Rdkit for peptide-based modeling, the Experimental-Torsion Basic Knowledge Distance Geometry algorithm for conformational search, MMFF94 for structure optimization, and Hdock for global search and hybrid strategy to dock molecules. Finally, PyMOL and LigPlot 2.1 were utilized for visualization.

### Transfection of IGF2BP3 small interfering RNA (siRNA)

IGF2BP3 siRNA and control siRNA dry powder were dissolved in RNase-free water to 20 µmol/L and stored at − 20 ℃. The Caco-2 cells were seeded at a density of 50% in the absence of antibiotics. Transfection complexes were formed using Opti-MEM, Lipofectamine RNAiMAX reagent (Thermo Scientific), and siRNA. Fresh medium was replaced after incubation for 8 h, and interference effects were detected after 72 h. The siRNAs are listed in Supplemental Table S3.

### RNA immunoprecipitation (RIP)

The RIP assay was performed according to the manufacturer’s instructions (Bersin Bio, Guangzhou, China). Briefly, at least 2 × 10^7^ Caco-2 cells were harvested and lysed. An IGF2BP3 antibody (2 µg) was added to the cell lysate, rotated overnight at 4 ℃, and immobilized on magnetic beads at 4 ℃ for 1 h. After washings for five times, the beads were treated with proteinase K at 55 ℃ for 1 h. The co-precipitated RNAs were used for qPCR to calculate the relative enrichment.

### RNA pulldown assay

The RNA pulldown assay was performed according to the manufacturer’s instructions (Bersin Bio). A biotin-labeled CLDN11 full-length probe was synthesized (Focobio, Guangzhou, China). A positive probe (Focobio), which binds to HuR (an RNA-binding protein commonly expressed in various cells) was used, while a negative probe (Focobio) served as a control. The probes were combined with streptavidin-labeled magnetic beads and mixed with Caco-2 cell lysis buffer for 2 h. The magnetic beads were collected and treated with an elution buffer. The collected proteins were identified by WB.

### CLDN11 mRNA stability

To determine the stability of CLDN11 mRNA under the downregulation of IGF2BP3, Caco-2 cells were treated with 10 µg/mL actinomycin D (Abmole Bioscience, Texas, USA) for 0, 3, 6, and 9 h. Samples were collected on time, and RNA extraction and qPCR were performed. The expression of CLDN11 mRNA was calculated and normalized to β-actin.

### N6-methyladenosine(m6A) RNA methylation assessment

The m6A RNA methylation was detected according to the manufacturer’s instructions (EpigenTek, NewYork, USA). For this assay, 100 ng of total RNA was bound to assay wells and incubated at 37 ℃ for 90 min. Subsequently, m6A RNA capture antibody, detection antibody, enhancer solution, and fluorescence developer solution were added sequentially. Finally, relative fluorescence was measured and read on a fluorescence microplate reader (PerkinElmer) at 530_EX_/590_EM_ nm.

### Pro-inflammatory cytokines treatment in vitro and anti-TNFα therapy in vivo

The Caco-2 cells were seeded at a density of 3 × 10^5^ cells/well on 6-well plates and treated for TNF-α, IL-6, IL-1β, and IFN-γ for 24 h. Following treatment, the total RNA and proteins were collected from the cells for further analysis. One week before SAP induction, mice were intraperitoneally injected three times with 10 mg/kg infliximab (Meilunbio, Dalian, China) or isotype control IgG (Bio X cell, New Hampshire, USA).

### Statistical analysis

All statistical analyses were performed using SPSS Statistics software (version 27.0; IBM, Illinois, USA). A Student’s *t*-test was used to analyze the differences between the two groups. Data are presented as mean ± standard error of the mean. Statistical graphs were drawn using GraphPad Prism version 10.2 (GraphPad Software, California, USA).

### Role of the funding source

Funders of this study did not play any role in the study design, data collection and analyses, or writing.

## Results

### Increased intestinal permeability and downregulation of CLDN11 during experimental obesity-related SAP

After feeding with a high-fat diet for 4 months, compared with the control group, body weight (Supplemental Fig. S1A) and serum TG and TC levels (Supplemental Fig. S1B) of obese group were significantly higher. Mice fed a regular diet and a high-fat diet were divided into two subgroups, totaling four groups: normal-weight mice treated with saline (NC), normal-weight mice induced by SAP (NS), obese mice treated with saline (FC), and obese mice induced by SAP (FS). After 24 h of intraperitoneal injection of CAE and LPS, serum AMY levels (Supplemental Fig. S1B) and pancreatic tissue inflammation damage scores (Supplemental Fig. S1C, D) in the FS and NS groups were higher than those in the FC and NC groups, respectively. And the pancreatic tissue inflammation damage scores in the FS group were higher than those in the NS group (Supplemental Fig. S1C, D). The FS group also observed an increase in pro-inflammatory cytokine levels, especially TNF-α (Supplemental Fig. S1E).

**Fig. 1:**
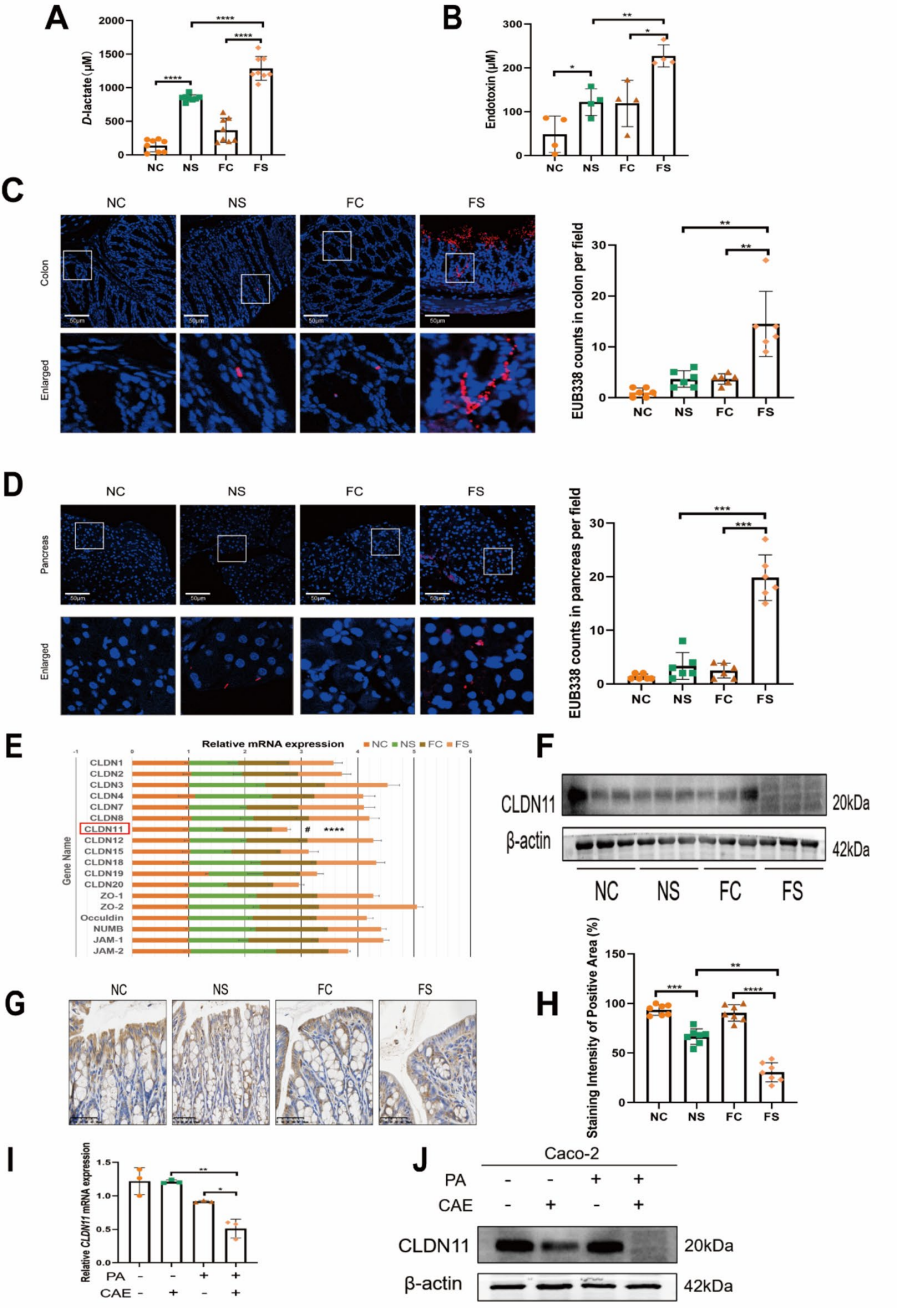
CLDN11 was decreased in the colon epithelium of obesity-related SAP. (**A**) *D*-lactate and (**B**) endotoxin levels in serum. (**A**) Each group *n* = 8. (**B**) Each group *n* = 4. (**C**) Representative FISH images of total bacteria detected by EUB338 probe in colon in 400× magnification. Each group *n* = 6. (**D**) Representative FISH images of total bacteria detected by EUB338 probe in pancreas in 400× magnification. Each group *n* = 6. DAPI (nucleus, blue fluorescence), EUB338 (total bacterial nucleic acid, red fluorescence) (**E**) mRNA expression analysis of mechanical intestinal barrier showed CLDN11 mRNA decreased in the obesity-related SAP mice model. ^#^NS vs. NC, ****FS vs. NS. NC *n* = 6, NS *n* = 7, FC *n* = 7, FS *n* = 9. (**F**) The relative protein levels of CLDN11 were detected by western blotting. Each group *n* = 3. (**G**) Representative IHC images of CLDN11-stained colon sections. (**H**) Statistical analysis on the staining area of CLDN11 among groups. Each group *n* = 7. The relative mRNA (**I**) and protein (**J**) levels of CLDN11 in Caco-2 and PANC-1 co-culture model that added CAE and PA. Each group *n* = 3. (*, ^#^*p* < 0.05, **p < 0.01, ***p < 0.001, ****p < 0.0001)

The indicators of intestinal permeability in the FS group, serum *D*-lactate (Fig. 1A), and endotoxin (Fig. 1B) levels were significantly higher than those in the other three groups. Bacterial translocation to the colon and pancreas of the FS group were also significantly increased (Fig. 1C, D & Supplemental Fig. S1F, G).

Based on the increased intestinal permeability in mice with obesity-related SAP and that the intestinal mechanical barrier is one of the most important defense lines against intestinal bacterial translocation, we examined the relative mRNA levels of multiple tight junction molecules in the colon of each group. The relative mRNA levels of CLDN11 were downregulated in the NS group, but were more severe in the FS group (Fig. 1E). The relative protein levels of CLDN11 were also reduced (Fig. 1F). IHC staining revealed that CLDN11 protein was mainly expressed in the epithelial and lamina propria layers of the colon (Fig. 1G). The expression of CLDN11 in the epithelial layer of the FS group showed an uneven decrease (Fig. 1H). In contrast, there was no difference in the expression of CLDN11 in the lamina propria among the four groups. The addition of CAE and PA to the Caco-2 and PANC-1 co-culture systems resulted in a decrease in the expression of CLDN11 (Fig. 1I, J) in Caco-2 cells. Therefore, we investigated the role and mechanism of CLDN11 in the intestinal epithelial cells of obesity-related SAP.

### CLDN11 regulated intestinal epithelial permeability in vivo and in vitro

**Fig. 2:**
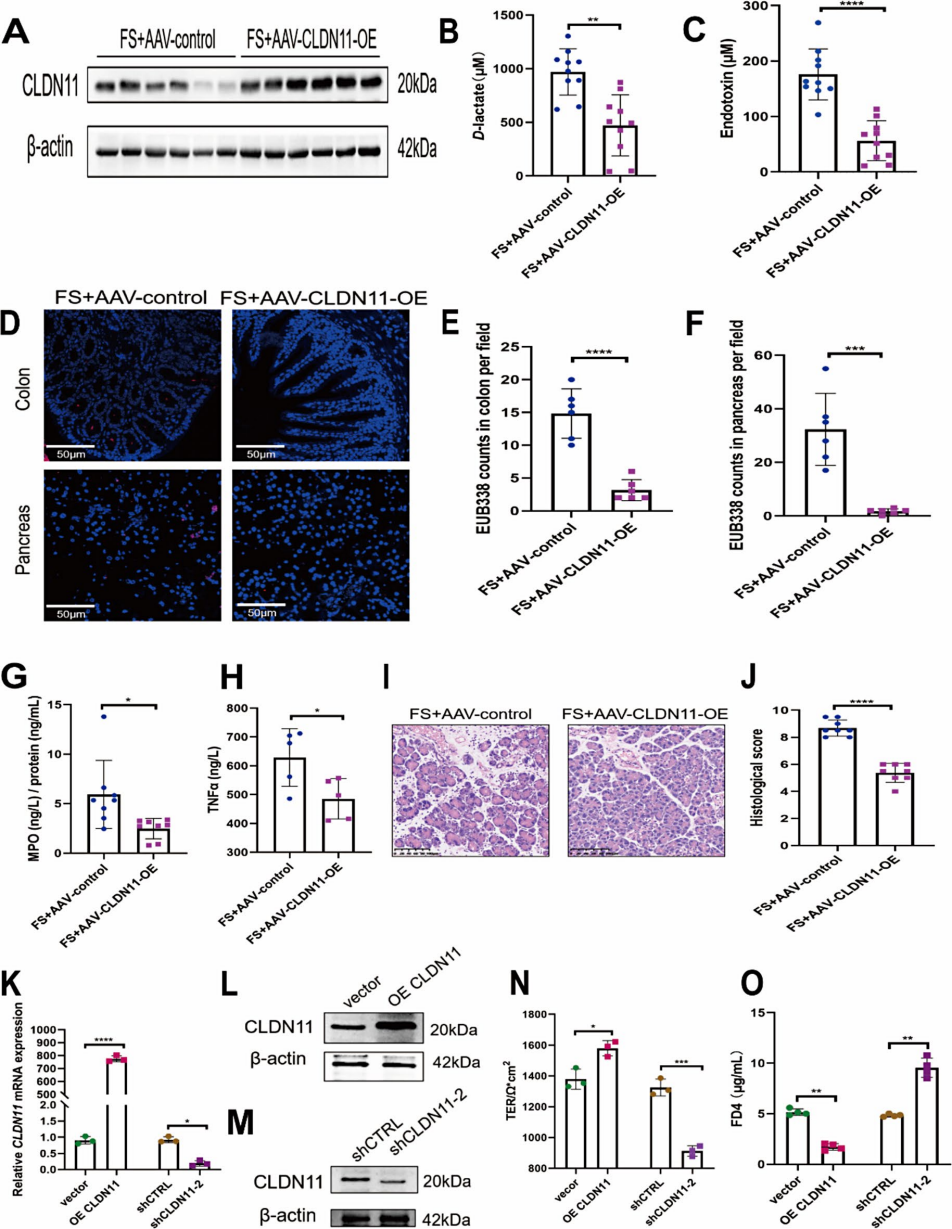
CLDN11 regulates intestinal permeability. (**A**) Western blotting of CLDN11 in the colon of AAV-CLDN11-OE mice and AAV-control mice. Each group *n* = 6. *D*-lactate (**B**) and endotoxin (**C**) levels in serum. Each group *n* = 10. (**D**) Representative FISH images of total bacteria detected by EUB338 probe. DAPI (nucleus, blue fluorescence), EUB338 (total bacterial nucleic acid, red fluorescence). EUB338 counts in colon (**E**) and pancreas (**F**) in 400× magnification per field were quantified. Each group *n* = 6. (**G**) MPO activities in pancreas. Each group *n* = 8. (**H**) Serum TNF-α levels. Each group *n* = 5. (**I**) Representative pancreatic histopathological images. (**J**) Histological scores. Each group *n* = 8. The relative mRNA (**K**) levels of CLDN11 in Caco-2 cells after overexpressing CLDN11 or CLDN11 knockdown. Each group *n* = 3. The relative protein levels of CLDN11 in Caco-2 cells after overexpressing CLDN11 (**L**) or CLDN11 knockdown (**M**). Each group *n* = 3. TER (**N**) and FD4 permeability (**O**) of OE CLDN11 or shCLDN11 stable Caco-2 monolayer cell model. Each group *n* = 3. (*p < 0.05, **p < 0.01, ***p < 0.001, ****p < 0.0001)

To understand the role of CLDN11 in the intestinal permeability of obesity-related SAP, we induced AAV-mediated overexpression of CLDN11. AAV serotype 9, combined with the broad-spectrum promoter CMV, was selected for its high transduction efficiency in intestinal epithelial cells. Mice were injected with AAV-CLDN11-OE or AAV-control (control vector) for transfection. To determine the efficiency of intestinal transfection, the expression of CLDN11 protein was examined (Fig. 2A, Supplemental Fig. S1H, I). AAV-CLDN11-OE mice with obesity-related SAP (FS + AAV-CLDN11-OE) had lower levels of serum *D*-lactate (Fig. 2B) and endotoxin (Fig. 2C) than AAV-control mice with obesity-related SAP (FS + AAV-control). Consistently, compared with the FS + AAV-control group, the FS + AAV-CLDN11-OE group showed significantly reduced bacterial translocation in the colon and pancreas (Fig. 2D–F). Additionally, lower levels of pancreatic MPO (Fig. 2G) and serum TNF-α (Fig. 2H) were detected in the FS + AAV-CLDN11-OE group. The pancreas of the FS + AAV-CLDN11-OE group showed an improvement in the pathological injury score (Fig. 2I, J).

We used a Caco-2 monolayer cell model and two detection methods, TER and FD4 permeability, to investigate whether CLDN11 regulates intestinal epithelial permeability. The OE CLDN11 and shCLDN11 cell lines were established (Supplemental Fig. S1J, K & Fig. 2K–M). Compared with the control group, the OE CLDN11 Caco-2 monolayer cells had higher TER (Fig. 2N) and lower FD4 permeability (Fig. 2O), whereas shCLDN11 Caco-2 monolayer cells had lower TER (Fig. 2N) and higher FD4 permeability (Fig. 2O).

### IGF2BP3 protein bound to CLDN11 mRNA and regulated intestinal epithelial barrier permeability

**Fig. 3:**
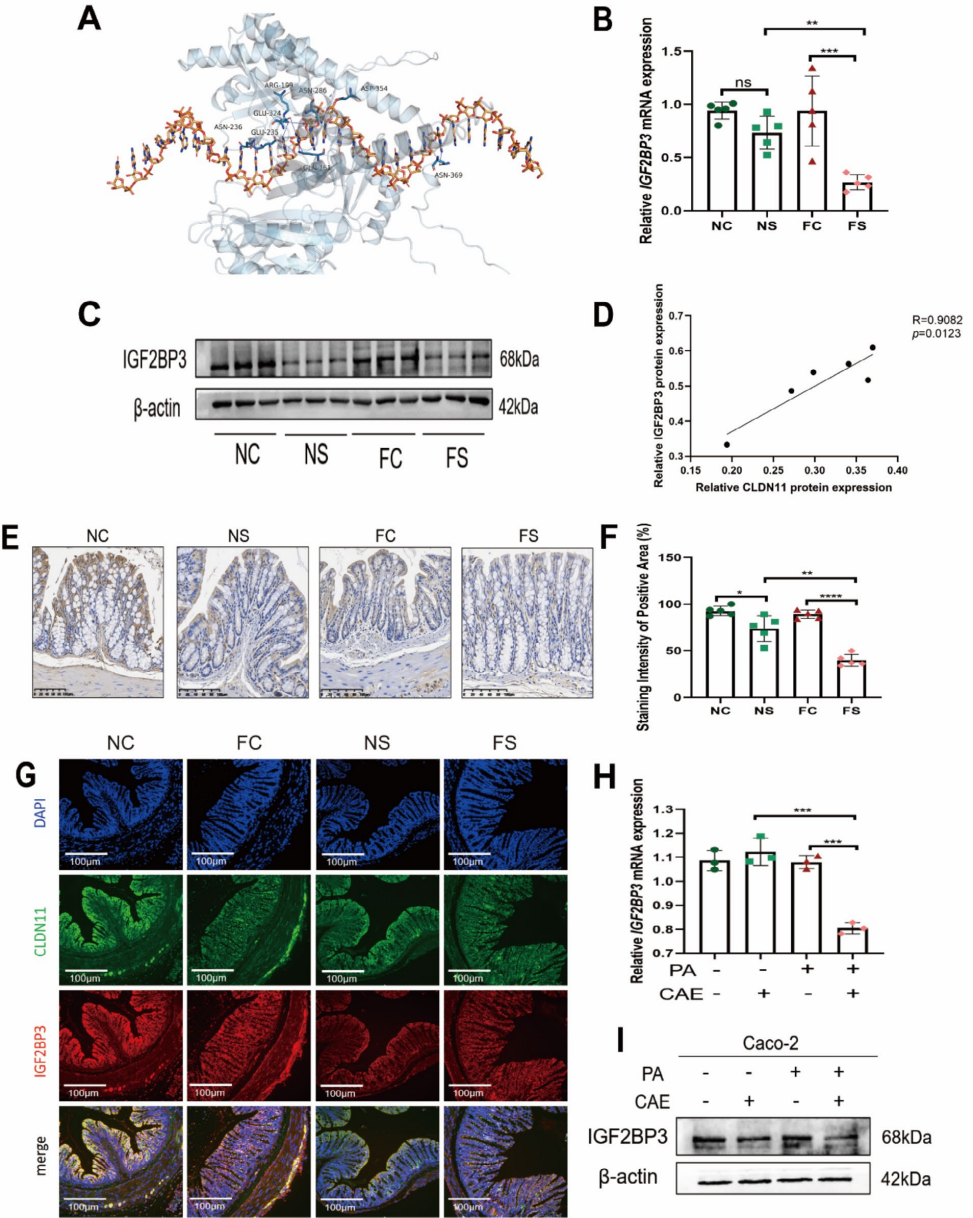
IGF2BP3 was decreased in the colon epithelium of obesity-related SAP. (**A**) Molecular docking between IGF2BP3 protein and CLDN11 mRNA. (**B**) The relative mRNA levels of IGF2BP3 in the obesity-related SAP mice model. Each group *n* = 5. (**C**) The relative protein levels of CLDN11 were detected by western blotting. Each group *n* = 3. (**D**) Spearman’s correlation analysis between the relative protein levels of IGF2BP3 and CLDN11. *n* = 6. (**E**) Representative IHC images of IGF2BP3-stained colon sections. (**F**) Statistical analysis on the staining area of IGF2BP3 among groups. Each group *n* = 5. (**G**) IF showed expression and colocalization of IGF2BP3 and CLDN11 in colon epithelium in 200× magnification per field. The relative mRNA (**H**) and protein (**I**) levels of IGF2BP3 in Caco-2 and PANC-1 co-culture model that added CAE and PA. Each group *n* = 3. (ns, no significance, *p < 0.05, **p < 0.01, ***p < 0.001, ****p < 0.0001)

To explore the mechanism of CLDN11 mRNA in obesity-related SAP, we utilized an online software tool, catRAPID, to predict protein-CLDN11 mRNA pairs. This tool identified several proteins were most likely to bind to CLDN11 mRNA, including IGF2BP3. We used bioinformatics techniques to predict the binding of IGF2BP3 protein and CLDN11 mRNA. Transcript 1 of CLDN11 mRNA bound well to IGF2BP3 protein (-220.59 kcal/mol) (Fig. 3A), while transcript 2 of CLDN11 mRNA was − 170 kcal/mol. Next, we verified the downregulation of IGF2BP3 in the colon tissues of patients with obesity-related SAP (Fig. 3B, C). As shown in Fig. 3D, the relative protein levels of IGF2BP3 were positively correlated with the relative protein levels of CLDN11 in the FS group. IHC analysis demonstrated that IGF2BP3 protein levels were reduced in epithelial cells of the colon (Fig. 3E, F). Both IGF2BP3 and CLDN11 were downregulated in the intestinal epithelium of patients with obesity-related SAP (Fig. 3G). Additionally, when CAE and PA were added to the co-culture system of Caco-2 and PANC-1 cells, the relative mRNA and protein levels of IGF2BP3 in Caco-2 decreased (Fig. 3H, I).

**Fig. 4:**
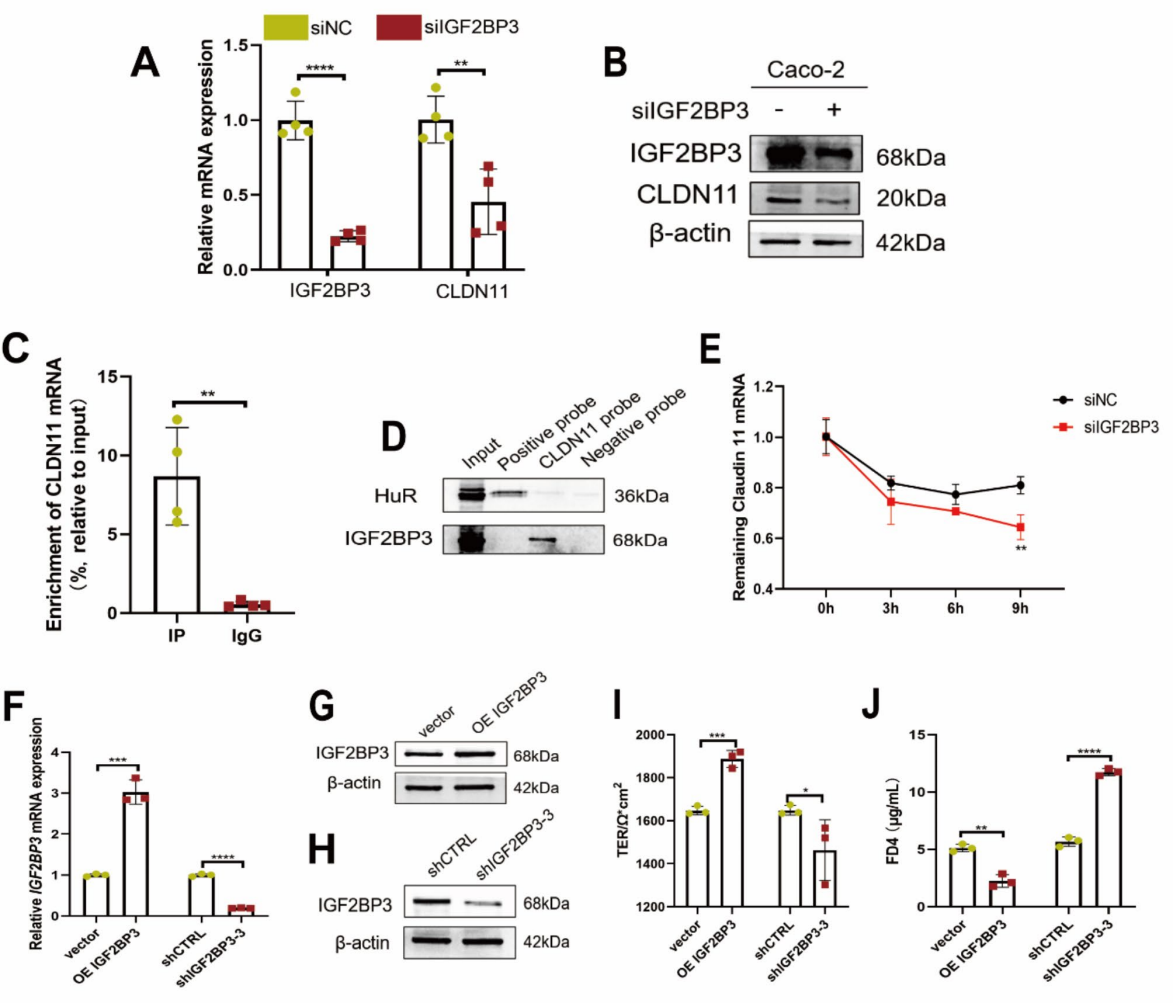
IGF2BP3 protein binds to CLDN11 mRNA and enhances the stability of CLDN11 mRNA. (**A**) CLDN11 mRNA decreased in Caco-2 cells after IGF2BP3 knockdown. Each group *n* = 4. (**B**) The relative protein levels of CLDN11 were detected by western blotting. IGF2BP3 bound to CLDN11 mRNA was confirmed by RIP-qPCR (**C**) and RNA pulldown (**D**). Each group *n* = 4. (**E**) The mRNA stability and degradation halftime of CLDN11 in Caco-2 cells treated by Actinomycin D. Each group *n* = 3. The relative mRNA (**F**) levels of IGF2BP3 in Caco-2 cells after overexpressing IGF2BP3 or IGF2BP3 knockdown. Each group *n* = 3. The relative protein levels of IGF2BP3 in Caco-2 cells after overexpressing IGF2BP3 (**G**) or IGF2BP3 knockdown (**H**). TER (**I**) and FD4 permeability (**J**) of OE IGF2BP3 or shIGF2BP3 stable Caco-2 monolayer cell model. Each group *n* = 3. (*p < 0.05, **p < 0.01, ***p < 0.001, ****p < 0.0001)

IGF2BP3 is an RNA-binding protein that stabilizes the translation of target mRNA. We validated in vitro whether IGF2BP3 protein can bind to and affect CLDN11 mRNA in intestinal epithelial cells. Our results showed that siIGF2BP3 significantly downregulates CLDN11 in Caco-2 cells (Fig. 4A, B). Direct binding between the IGF2BP3 protein and CLDN11 mRNA was further confirmed by RIP and RNA pulldown assays (Fig. 4C, D). In Caco-2 cells treated with actinomycin D, we observed that the stability of CLDN11 mRNA was significantly reduced when IGF2BP3 was knocked down compared to the control group (Fig. 4E). To further investigate, we established stable OE IGF2BP3 and shIGF2BP3 Caco-2 cells (Supplemental Fig. S2A, B & Fig. 4F–H). Compared to the control group, the OE CLDN11 Caco-2 monolayer cells had a higher TER (Fig. 4I) and lower FD4 permeability (Fig. 4J), whereas shCLDN11 Caco-2 monolayer cells had lower TER (Fig. 4I) and higher FD4 permeability (Fig. 4J).

### IGF2BP3 regulated CLDN11 mRNA in an RNA m6A-independent manner

**Fig. 5:**
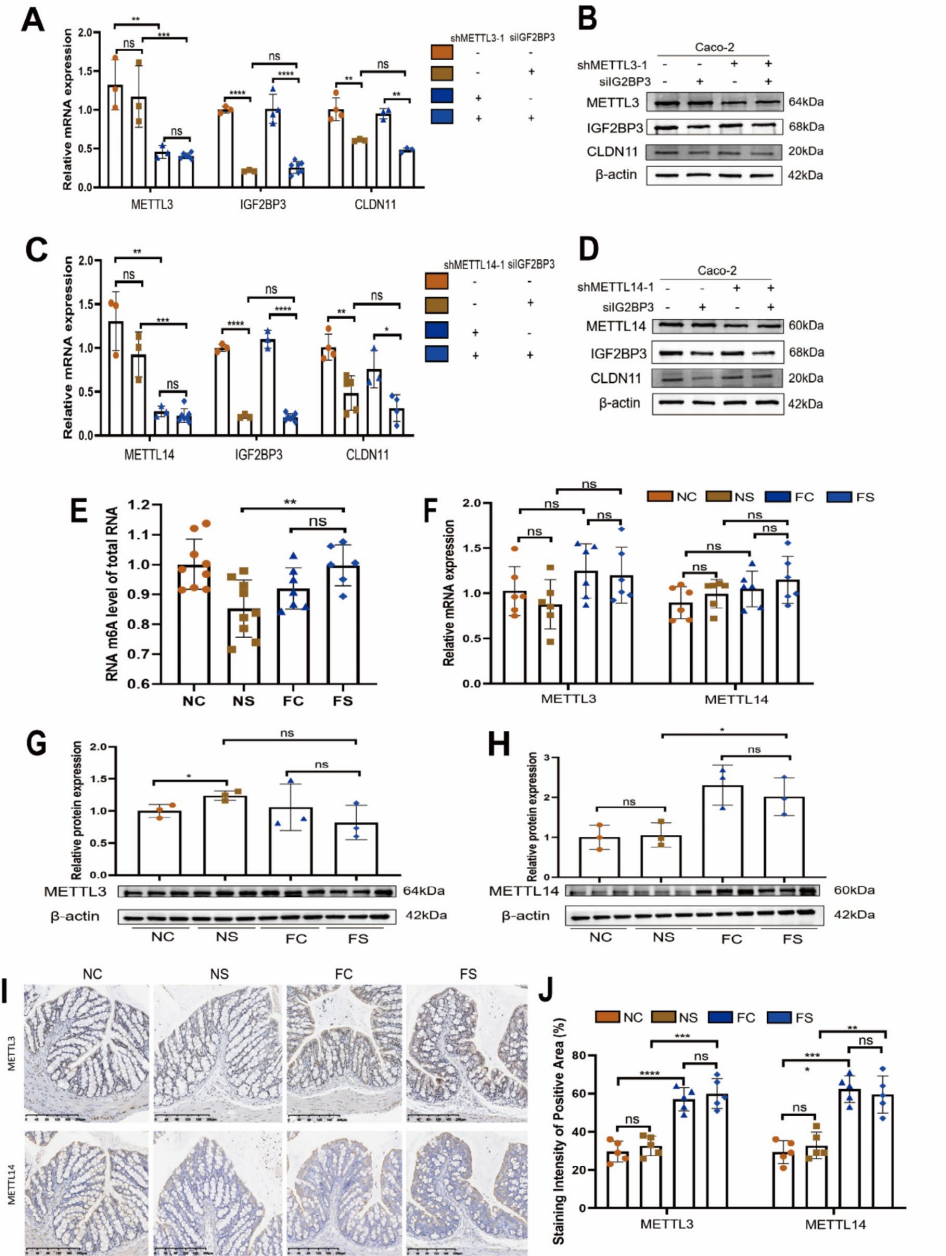
IGF2BP3 protein regulates CLDN11 mRNA by an RNA m6A independent manner. The relative mRNA (**A**) and protein (**B**) levels of METTL3, IGF2BP3 and CLDN11 in Caco-2 cells after METTL3 and IGF2BP3 knockdown. Each group *n* = 3. The relative mRNA (**C**) and protein (**D**) levels of METTL14, IGF2BP3 and CLDN11 in Caco-2 cells after METTL14 and IGF2BP3 knockdown. Each group *n* = 3. (**E**) RNA m6A level of total RNA in colon. NC *n* = 9, NS *n* = 9, FC *n* = 7, FS *n* = 6. (**F**) METTL3 and METTL14 mRNA show no difference in colon. Each group *n* = 6. The relative protein levels of METTL3 (**G**) and METTL14 (**H**) were detected by western blotting. Each group *n* = 3. (**I**) Representative IHC images of METTL3 and METTL14-stained colon sections. (**J**) Statistical analysis on the staining area of METTL3 and METTL14. Each group *n* = 5. (ns, no significance, *p < 0.05, **p < 0.01, ***p < 0.001, ****p < 0.0001)

IGF2BP3 demonstrated a high affinity for the m6A modification region of the target mRNA, and CLDN11 mRNA had abundant m6A modification sites (Supplemental Fig. S2C, D). To determine whether RNA m6A modification affects the binding of IGF2BP3 protein and CLDN11 mRNA, we established Caco-2 cells with m6A RNA “writer” METTL3 or METTL14 deletion (Supplemental Fig. S2E–I). Our results showed that knockdown of IGF2BP3 in these two cell lines resulted in the downregulation of CLDN11, indicating that the interaction of IGF2BP3 protein and CLDN11 mRNA was independent of METTL3 (Fig. 5A, B) or METTL14 (Fig. 5C, D). Moreover, there was no significant difference in RNA m6A levels in the colon tissues of the obesity-related SAP mice (Fig. 5E). Similarly, there were no differences in the relative mRNA (Fig. 5F) and protein (Fig. 5G, H) levels of METTL3 and METTL14 between the groups. IHC staining showed that METTL3 and METTL14 were mainly expressed in the epithelium of the colon (Fig. 5I). There was no difference in the expression of METTL3 and METTL14 among the four groups (Fig. 5J).

### TNF-α interfered with IGF2BP3-stabilized CLDN11 mRNA

**Fig. 6:**
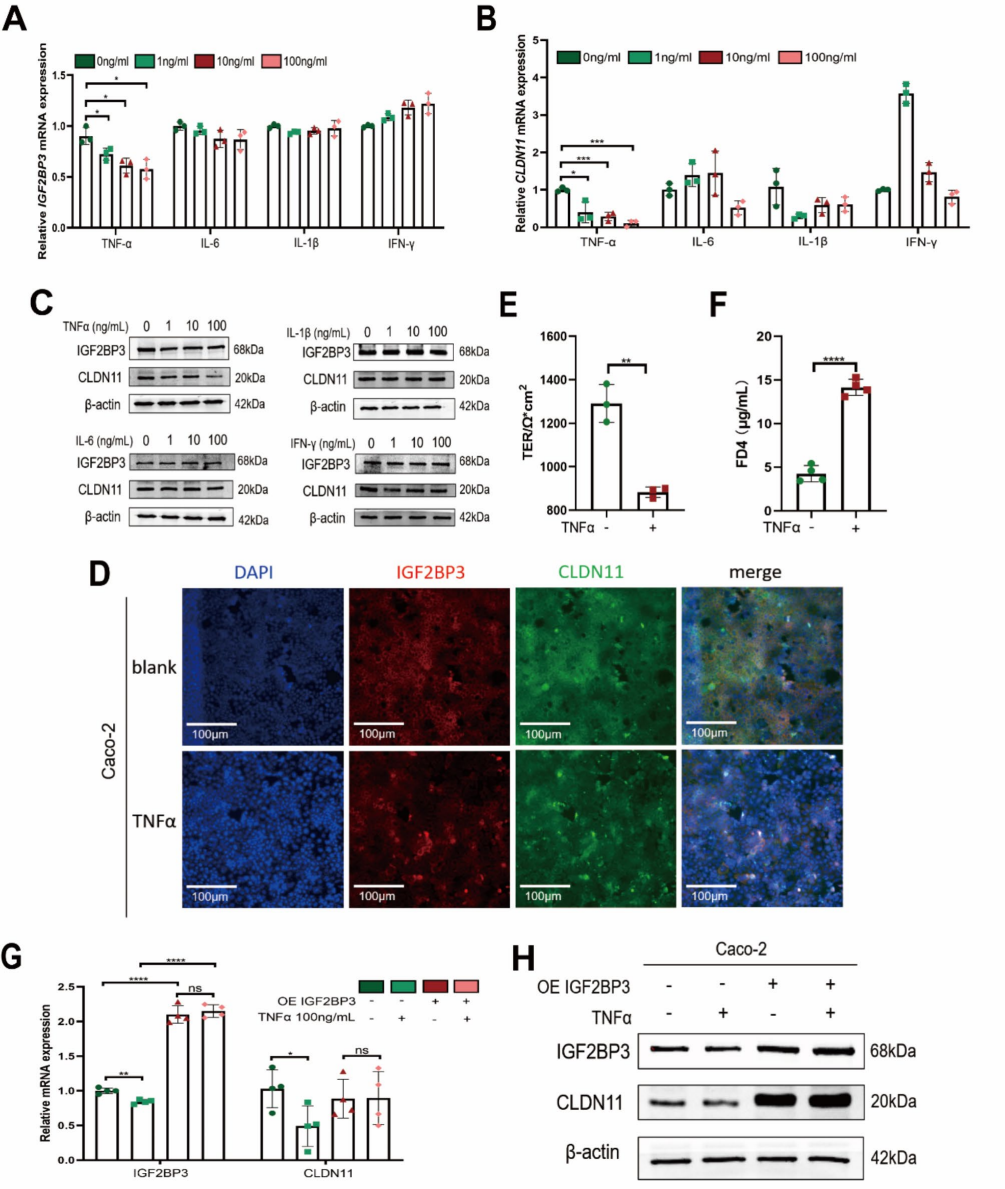
The expression of CLDN11 was mediated by TNF-α through IGF2BP3 in Caco-2 cells. Only TNF-α treatment led to decrease in the relative mRNA levels of IGF2BP3 (**A**) and CLDN11 (**B**). Each group *n* = 3. (**C**) The relative protein levels of IGF2BP3 and CLDN11 were detected by western blotting. (**D**) Representative IF images of Caco-2 cells mediated by 100ng/mL TNF-α in 200× magnification per field. TER (**E**) and FD4 permeability (**F**) of Caco-2 monolayer cell model treated with 100ng/mL TNF-α. The relative mRNA (**G**) and protein (**H**) levels of CLDN11 in OE IGF2BP3 stable Caco-2 cells mediated by 100ng/mL TNF-α. Each group *n* = 4. (ns, no significance, *p < 0.05, **p < 0.01, ***p < 0.001, ****p < 0.0001)

To determine which inflammatory cytokine contributed to the decreased IGF2BP3 expression, we treated Caco-2 cells with intestinal epithelium-relevant cytokines (TNF-α, IL-6, IL-1β, and IFN-γ). Only TNF-α treatment significantly downregulated the expression of IGF2BP3 and CLDN11 in a dose-dependent manner (Fig. 6A–C). Treating Caco-2 cells with TNF-α (100 ng/mL) for 48 h greatly decreased the expression of IGF2BP3 and CLDN11 (Fig. 6D). Moreover, treating the Caco-2 monolayer cell model with TNF-α (100 ng/mL) for 48 h resulted in lower TER (Fig. 6E) and higher FD4 permeability (Fig. 6F).

However, studies have reported that TNF-α can directly induce intestinal barrier defects, such as downregulating CLDN1, 5, 7 (Amasheh et al. 2009). We further clarified whether the downregulation of CLDN11 under TNF-α treatment was directly caused by IGF2BP3. Following TNF-α treatment of stable cell lines overexpressing IGF2BP3, the expression of IGF2BP3 was not downregulated, nor was the expression of CLDN11 (Fig. 6G, H).

### Anti-TNFα therapy reduced high intestinal permeability and alleviated inflammation in obesity-related SAP

**Fig. 7:**
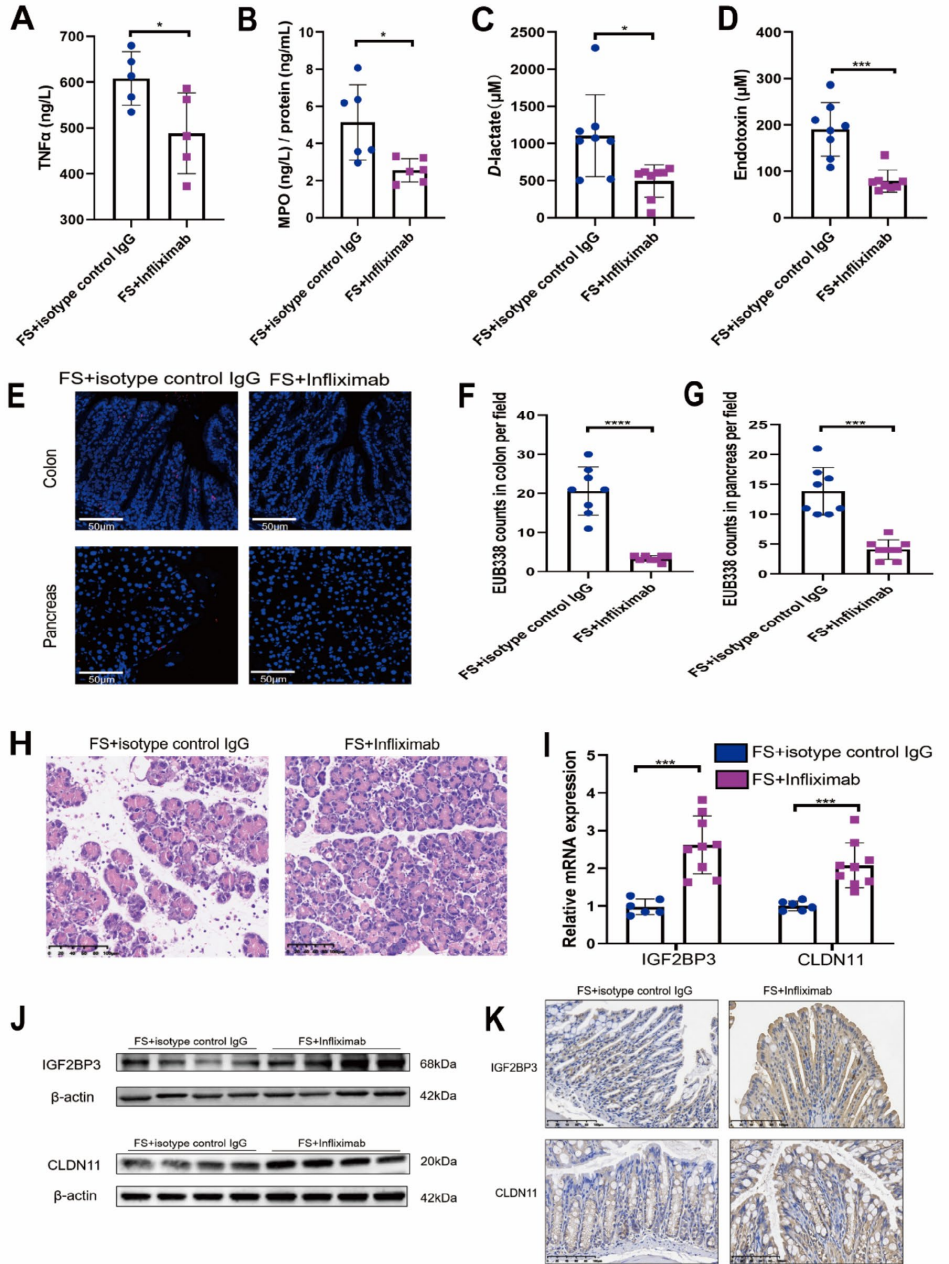
Anti-TNFα therapy improved intestinal permeability and alleviates pancreatic inflammation in obesity-related SAP mice. (**A**) Serum TNF-α levels. Each group *n* = 5. (**B**) MPO activities in pancreas. Each group *n* = 6. *D*-lactate (**C**) and Endotoxin (**D**) levels in serum. Each group *n* = 8. (**E**) Representative FISH images of total bacteria detected by EUB338 probe. DAPI (nucleus, blue fluorescence), EUB338 (total bacterial nucleic acid, red fluorescence). EUB338 counts in colon (**F**) and pancreas (**G**) in 400× magnification per field were quantified. Each group *n* = 8. (**H**) Representative pancreatic histopathological images. The relative mRNA (**I**) and protein (**J**) levels of IGF2BP3 and CLDN11. (I) FS + isotype control IgG *n* = 6, FS + Infliximab *n* = 9. (J) Each group *n* = 4. (**K**) Representative IHC images of IGF2BP3, CLDN11-stained colon sections. (*p < 0.05, ***p < 0.001, ****p < 0.0001)

Few measures against pro-inflammatory cytokines are administered in treating obesity-related SAP; thus, we applied anti-TNFα therapy in mice models of obesity-related SAP. The effectiveness of anti-TNFα therapy was first confirmed (Fig. 7A). Pancreatic MPO levels decreased (Fig. 7B), and the FS + Infliximab group had lower serum *D*-lactate (Fig. 7C) and endotoxin (Fig. 7D) levels than the FS + isotype control IgG group. Compared to the FS + isotype control IgG group, the FS + Infliximab group showed decreased bacterial translocation to the colon and pancreas (Fig. 7E–G). In addition, the histological scores of the pancreas in the FS + Infliximab group were lower (Fig. 7H, Supplemental Fig. S2J). We also found that anti-TNFα therapy improved intestinal permeability while simultaneously upregulating the expression of IGF2BP3 and CLDN11 (Fig. 7I–K and Supplemental Fig. S2K).

## Discussion

In our study, obesity exacerbated SAP by increasing intestinal permeability and bacterial translocation, with CLDN11 as a key molecule in increasing intestinal permeability. The increased exogenous expression of CLDN11 in intestinal epithelial cells helps maintain intestinal barrier integrity and alleviates SAP. Bioinformatics tools were used to predict whether the IGF2BP3 protein can bind to CLDN11 mRNA in intestinal epithelial cells. RIP and RNA pull-down assays demonstrated the interaction between CLDN11 mRNA and IGF2BP3 protein. Under the effect of TNF-α in obesity-related SAP, the transcription and translation of IGF2BP3 in intestinal epithelial cells were inhibited, thereby affecting the expression of CLDN11. Administration of antiTNFα therapy significantly improved intestinal permeability and pancreatitis and upregulated the IGF2BP3 and CLDN11 expression. Overall, the TNF-α/IGF2BP3/CLDN11 axis was involved in the mechanism of obesity-mediated SAP exacerbation and suggests potential therapeutic targets.

CLDN11 is a member of the classic Claudin family and primarily plays a role in tight junctions. It has been found that CLDN11 was downregulated in the blood testis barrier (Kato et al. 2020), blood-brain barrier (Uchida et al. 2019), blood-spinal cord barrier (Uchida et al. 2019), blood-arachnoid barrier (Uchida et al. 2019), and venous endothelial barrier (Li et al. 2017). The reduction in CLDN11 impaired the integrity of barriers, altered barrier function, and caused different types and degrees of pathological changes. Our findings elucidate the role of CLDN11 in the intestinal barrier, further demonstrating the importance of CLDN11 in the internal barrier and its potential as a target for barrier deficiency diseases.

In previous studies, regulatory mechanisms of CLDN11 expression involve DNA and transcription levels. Abe et al. (Abe et al. 2016) reported that CLDN11 was silenced owing to the high methylation of its promoter. In contrast, Li et al. (Li et al. 2017) found that increased acetylation of histone H3K9 in the promoter of the CLDN11 gene activated its transcription. Overexpression of the transcription factor Snail upregulates CLDN11 (Li et al. 2019). In the seminiferous epithelium, changes in the ratio of positive regulatory transcription factors (GATA, nuclear factor YA, and cAMP response element-binding protein) to negative factor (Smads) controlled the transcription of CLDN11 (Liu et al. 2007). MiRNA-421 (Yang et al. 2017), miRNA-92a-3p (Yamada et al. 2019), and miRNA-99b (Yang et al. 2015) targeting CLDN11 mRNA mediated its degradation. We discovered an unreported CLDN11 regulatory mechanism that involved the binding of the IGF2BP3 protein to CLDN11 mRNA and the regulation of CLDN11 mRNA stability. Owing to IGF2BP3 being a “reader” of RNA m6A (Huang et al. 2020), we also revealed that the binding of IGF2BP3 protein to CLDN11 mRNA was not dependent on m6A modification despite its abundant RNA m6A sites. This discovery elucidated the regulatory mechanism of the key barrier molecule, CLDN11, which is beneficial for optimizing the treatment of barrier deficiency diseases.

The expression of IGF2BP3 is regulated by various mechanisms in different pathological conditions. Long noncoding RNA (lncRNA) such as metastasis-associated lung adenocarcinoma transcript 1 (MALAT1) (Tian et al. 2023) and DNA methylation–deregulated and RNA m6A reader–cooperating lncRNA (DMDRMR) (Gu et al. 2021) directly bound with IGF2BP3 protein and regulated its expression in diabetic retinopathy and clear cell renal cell carcinoma, respectively. Moreover, circRNAs such as circ-transportin 3 (TNPO3) (Yu et al. 2021) and circular nuclear factor of activated T cells 3 (circNFATC3) (Yang et al. 2023) interacted with IGF2BP3 protein and further regulated its expression in gastric cancer. A phosphatase and kinase complexes regulated the transcription of IGF2BP3 (Zhang et al. 2023). Huang et al. (Huang et al. 2024) found that the expression of IGF2BP3 significantly decreased during adipogenic differentiation of mesenchymal stem cells. All above, the expression of IGF2BP3 is finely regulated through various mechanisms, which operated at the levels of gene transcription and protein interaction, and are also influenced by environment and endogenous signals.

During the development of AP, many cytokines such as TNF-α, IL-1β, IL-6, and IFN-γ were released, further promoting tissue damage (Gukovskaya et al. 2017; McKay et al. 1996). Cytokines can bind to cell-surface receptors, ultimately affecting the expression of several genes. We found that the levels of TNF-α in experimental obesity-related SAP were higher than IL-1β, IL-6, and IFN-γ, indicating its potential unique role. TNF-α activates signaling pathways leading to NF-κB and activating protein-1 (AP-1) through two distinct receptors, tumor necrosis factor receptor 1 (TNFR1) and TNFR2 (Baud et al. 2001). NF-κB and AP-1 are key transcription factors that play important roles in TNF-α-induced gene expression (Baud et al. 2001). In this study, we treated intestinal epithelial cells with TNF-α in vitro and found a decrease in the expression of IGF2BP3 and CLDN11, suggesting that TNF-α is a regulatory factor of IGF2BP3 and CLDN11. Furthermore, it was found that the expression of CLDN11 downregulated by TNF-α was dependent on IGF2BP3, rather than being directly interfered with by TNF-α. Therefore, TNF-α, IGF2BP3, and CLDN11 is a regulatory mechanism.

In clinical practice, to improve intestinal barrier function, doctors often recommend early enteral nutrition to reduce bacterial translocation, thus reducing the risk of SAP (Hines et al. 2019). In this study, we applied anti-TNFα therapy aimed at targeting the elevated TNF-α in obesity-related SAP. Anti-TNFα therapy has long been carried out in the clinical treatment of autoimmune diseases such as Crohn’s disease (Peyrin-Biroulet et al. 2008) and rheumatoid arthritis (Bazzani et al. 2009). Recently, anti-TNFα therapy is being evaluated in other diseases, including coronavirus disease 2019, neuropsychiatric diseases and cancers (Leone et al. 2023). After administrating anti-TNFα therapy, we observed significant improvements in intestinal permeability and pancreatitis, indicating that it may be a potential therapeutic approach for improving intestinal permeability. However, we lacked direct evidence to suggest whether anti-TNFα therapy improved intestinal permeability by rescuing IGF2BP3 and CLDN11. Simultaneously, adverse effects of anti-TNFα therapy, such as infection and malignant tumors, cannot be ignored. However, we did not investigate these adverse effects. Further research is needed on how anti-TNFα therapy relieves SAP and how to use it safely to maintain the intestinal barrier of SAP.

**Fig. 8:**
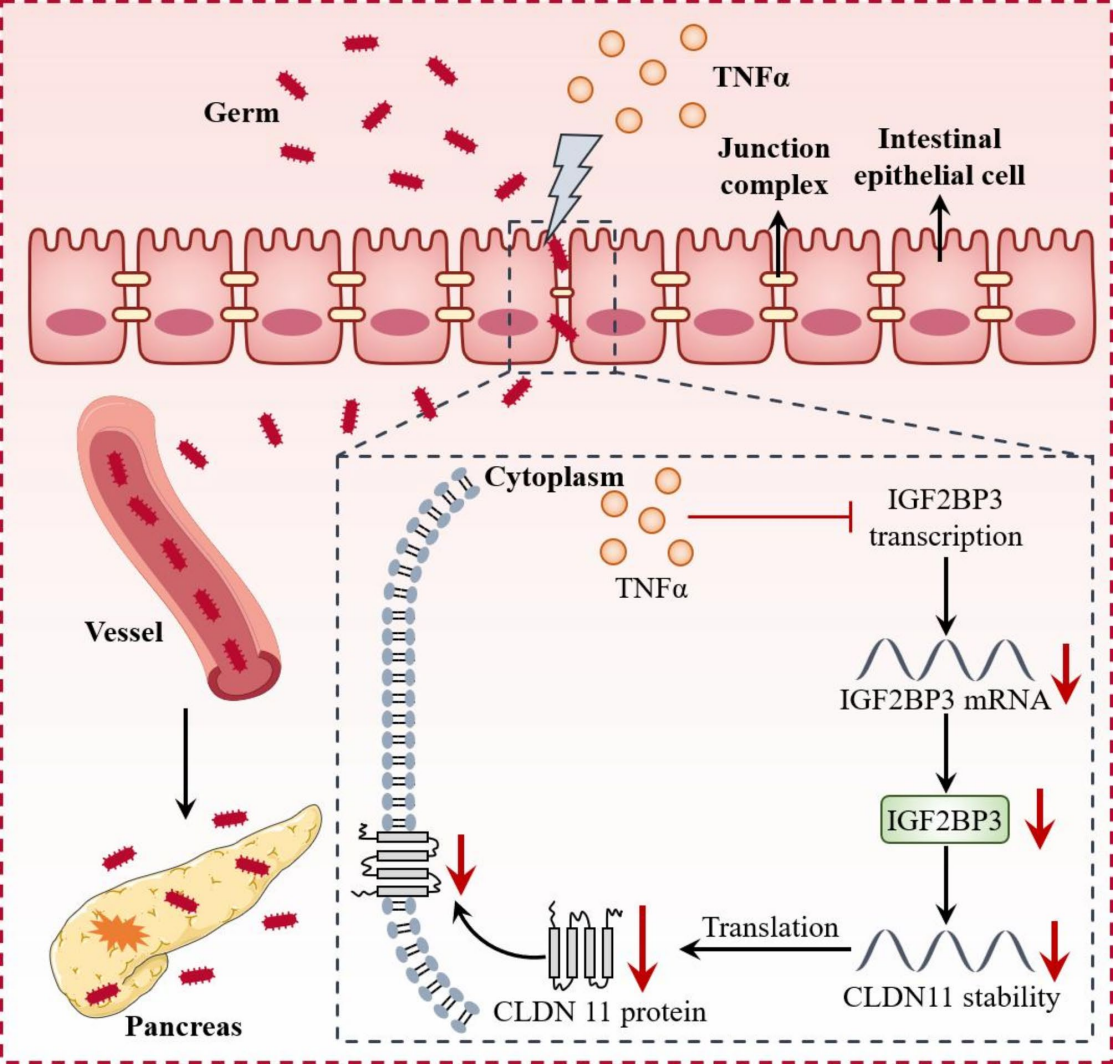
CLDN11 maintains intestinal permeability and TNF-α disrupts IGF2BP3-stabilized CLDN11 mRNA in obesity-related severe acute pancreatitis. CLDN11 is downregulated and regulates intestinal permeability during experimental obesity-related SAP. IGF2BP3 regulates the stability of CLDN11 mRNA, while TNF-α inhibits the IGF2BP3/CLDN11 axis, which explains the downregulation of CLDN11 and increased intestinal permeability in obesity-related SAP.

Overall, an increase in TNF-α impaired the stability of IGF2BP3-dependent CLDN11 mRNA in obesity-related SAP, aggravating intestinal permeability and pancreatic inflammation. Anti-TNFα therapy may be an optional therapy for obesity-related SAP. (Fig. 8).

## Electronic supplementary material

Below is the link to the electronic supplementary material.

Supplementary Material 1

Supplementary Material 2

## Data Availability

The datasets that support the findings of this study are available from the corresponding authors on reasonable request.

## Abbreviations

AP: Acute pancreatitis
SAP: Severe acute pancreatitis
CLDN11: Claudin 11
IGF2BP3: Insulin-like growth factor 2 mRNA-binding protein 3
TG: Triglyceride
TC: Total cholesterol
CAE: Caerulein
LPS: Lipopolysaccharide
AMY: Amylase
TNF-α: Tumor necrosis factor-α
IHC: Immunohistochemistry
PA: Palmitic acid
AAV: Adeno-Associated Virus
MPO: Myeloperoxidase
TER: Transepithelial resistance
FD4: Fluorescein isothiocyanate-dextran 4 kDa
METTL3: Methyltransferase like protein 3
METTL14: Methyltransferase like protein 14
m6A: N6-methyladenosine
IL-6: Interleukin-6
IL-1β: Interleukin − 1β
IFN-γ: Interferon-γ
WB: Western blotting
shRNAs: Short hairpin RNAs
siRNA: Small interfering RNA
