# Supplementary material for: Disrupting of IGF2BP3-stabilized CLDN11 mRNA by TNF-α increases intestinal permeability in obesity-related severe acute pancreatitis
Source: Mol Med. 2025 Jan 24;31:24. doi: 10.1186/s10020-025-01078-9 (PMC11762095; doi:10.1186/s10020-025-01078-9)
Supplement: Supplementary file 1 — Supplementary Material 1 [file 10020_2025_1078_MOESM1_ESM.docx]

**Supplemental Table 1** Primer sequences used in qPCR experiments

| Primer name | Primer sequence |
| --- | --- |
| mouse-β-actin | Forward: GGCTGTATTCCCCTCCATCG |
|  | Reverse: CCAGTTGGTAACAATGCCATGT |
| mouse-Claudin 1 | Forward: AGGTCTGGCGACATTAGTGG |
|  | Reverse: CGTGGTGTTGGGTAAGAGGT |
| mouse-Claudin 2 | Forward: AGTGGCTGTAGTGGGTGGAG |
|  | Reverse: AAAGGATGACTCCGGCTACC |
| mouse-Claudin 3 | Forward: GAGATGGGAGCTGGGTTGTA |
|  | Reverse: GTAGTCCTTGCGGTCGTAGG |
| mouse-Claudin 4 | Forward: GGGAATCTCCTTGGCAGTCC |
|  | Reverse: CGATGTTGCTGCCGATGAAG |
| mouse-Claudin 7 | Forward: AGCATGTTCCTGGATTGGTC |
|  | Reverse: CTCCCCAGCTCACACGTATT |
| mouse-Claudin 8 | Forward: GCAACCTACGCTCTTCAAATGG |
|  | Reverse: TTCCCAGCGGTTCTCAAACAC |
| mouse-Claudin 11 | Forward: ATGGTAGCCACTTGCCTTCAG |
|  | Reverse: AGTTCGTCCATTTTTCGGCAG |
| mouse-Claudin 12 | Forward: CTTCCTGTGTGGTATTGCCTC |
|  | Reverse: AAATCGTCAGGTTCTTCTCGTTT |
| mouse-Claudin 15 | Forward: ATGTCGGTAGCTGTGGAGAC |
|  | Reverse: GGACGGAAAGTCCCAGCAG |
| mouse-Claudin 18 | Forward: GTGGAGCACTCAAGACCTGTA |
|  | Reverse: CGATCATCAGGGCTCGTACAG |
| mouse-Claudin 19 | Forward: CAGTCTTCCTATGCAGGCGAT |
|  | Reverse: GTGCTGACTGGATATGACCGT |
| mouse-Claudin 20 | Forward: CTGGGATGTTTAGCTGCACTC |
|  | Reverse: GCAGTTCATTCCTGCTATCGAA |
| mouse-ZO-1 | Forward: GACCTTGATTTGCATGACGA |
|  | Reverse: AGGACCGTGTAATGGCAGAC |
| mouse-ZO-2 | Forward: CAGTCCCTATGCCTGAGAGC |
|  | Reverse: TTGGAACCGCATAGATGTCA |
| mouse-Occludin | Forward: ACACTTGCTTGGGACAGAGG |
|  | Reverse: AAGGAAGCGATGAAGCAGAA |
| mouse-NUMB | Forward: GCAGATGGACTCAGAGTTGTGGA |
|  | Reverse: GTGCCATCACGGCATATGTAAGA |
| mouse-JAM-1 | Forward: TCTCTTCACGTCTATGATCCTGG |
|  | Reverse: TTTGATGGACTCGTTCTCGGG |
| mouse-JAM-2 | Forward: GATCGTCGCCCTGGACTATC |
|  | Reverse: GTGACTTCTTGACGGTGGTCT |
| mouse-IGF2BP3 | Forward: GTATGGAGTGGTGGAGAG |
|  | Reverse: TCCTTACTGGAATAGGTTACAT |
| mouse-METTL3 | Forward: GCTTCGCGAGAGATTGCAG |
|  | Reverse: TAGGCACGGGACTATCACTAC |
| mouse-METTL14 | Forward: GGGAAAGAAACCGATCCAATTT |
|  | Reverse: AGTAAAGCCGCCTCTGTG |
| human-β-actin | Forward: ACCTTCTACAATGAGCTGCG |
|  | Reverse: CCTGGATAGCAACGTACATGG |
| human-Claudin 11 | Forward: GTGACCACCTCCACCAATGAC |
|  | Reverse: CAGGGGCTTGCAGTGGTACA |
| human-IGF2BP3 | Forward: GAGGCGCTTTCAGGTAAAATAG |
|  | Reverse: AATGAGGCGGGATATTTCGTAT |
| human-METTL3 | Forward: TTGTCTCCAACCTTCCGTAGT |
|  | Reverse: CCAGATCAGAGAGGTGGTGTAG |
| human-METTL14 | Forward: AGAAACTTGCAGGGCTTCCT |
|  | Reverse: TCTTCTTCATATGGCAAATTTTCTT |

Abbreviations: ZO-1, zonula occludens 1; ZO-2, zonula occludens 2; JAM-1, junctional adhesion molecule 1; JAM-2, junctional adhesion molecule 2; IGF2BP3, insulin-like growth factor 2 mRNA-binding protein 3; METTL3, methyltransferase like protein 3; METTL14, methyltransferase like protein 14.

**Supplemental Table 2** Antibodies used in western blotting, immunohistochemistry and immunofluorescence experiments

| Antibodies name | Antibodies source | Antibodies usage |
| --- | --- | --- |
| CLDN11 | Thermo Science (36-4500) | 1:100 for IHC&IF;  1:1000 for WB |
| IGF2BP3 | Santa (sc-390639) | 1:275 for IHC&IF;  1:1000 for WB |
| β-actin | Proteintech (20536-1-AP) | 1:5000 for WB |
| METTL3 | Proteintech (80323-1-RR) | 1:275 for IHC;  1:5000 for WB |
| METTL14 | Proteintech (80790-1-RR) | 1:275 for IHC;  1:3000 for WB |
| HRP-conjugated affinipure goat anti-rabbit IgG | Proteintech (SA00001-2) | 1:5000 for WB |
| HRP-conjugated affinipure goat anti-mouse IgG | Proteintech (SA00001-1) | 1:5000 for WB |
| Multi-rAb CoraLite^®^ Plus 555-Goat anti-rabbit recombinant secondary antibody | Proteintech (RGAR003) | 1:500 for IF |
| Multi-rAb CoraLite^®^ Plus 647-Goat anti-mouse recombinant secondary antibody | Proteintech (RGAM005) | 1:500 for IF |

Abbreviations: CLDN11, claudin 11; IGF2BP3, insulin-like growth factor 2 mRNA-binding protein 3; METTL3, methyltransferase like protein 3; METTL14, methyltransferase like protein 14; IHC, immunohistochemistry; IF, immunofluorescence; WB, western blotting.

**Supplemental Table 3** shRNAs and siRNA sequences

| sequences name | sequence |
| --- | --- |
| shCLDN11-1 | GGTGTAAACACTGATGGTATACTCGAGTATACCATCAGTGTTTACACC |
| shCLDN11-2 | GAGACCACCATCGTGAGCTTTCTCGAGAAAGCTCACGATGGTGGTCTC |
| shCLDN11-3 | GAAGAGTGCCCACGTATAAGACTCGAGTCTTATACGTGGGCACTCTTC |
| shIGF2BP3-1 | CGGTGAATGAACTTCAGAATTCTCGAGAATTCTGAAGTTCATTCACCG |
| shIGF2BP3-2 | GCAGGAATTGACGCTGTATAACTCGAGTTATACAGCGTCAATTCCTGC |
| shIGF2BP3-3 | GCTGCTGAGAAGTCGATTACTCTCGAGAGTAATCGACTTCTCAGCAGC |
| shMETTL3-1 | GGAGATCCTAGAGCTATTATTCAAGAGATAATAGCTCTAGGATCTCC |
| shMETTL3-2 | GCCTTAACATTGCCCACTGATCTCGAGATCAGTGGGCAATGTTAAGGC |
| shMETTL3-3 | GCCAAGGAACAATCCATTGTTCTCGAGAACAATGGATTGTTCCTTGGC |
| shMETTL14-1 | CCATGTACTTACAAGCCGATACTCGAGTATCGGCTTGTAAGTACATGG |
| shMETTL14-2 | GCATTGGTGCCGTGTTAAATACTCGAGTATTTAACACGGCACCAATGC |
| shMETTL14-3 | GCTGGACTTGGGATGATATTACTCGAGTAATATCATCCCAAGTCCAGC |
| shCTRL | CCTAAGGTTAAGTCGCCCTCGCTCGAGCGAGGGCGACTTAACCTTAGG |
| IGF2BP3 siRNA | GCAGGAAUUGACGCUGUAUAAdTdT |
| control siRNA | UUCUCCGAACGUGUCACGUdTdT |

Abbreviations: shRNAs, short hairpin RNAs; siRNA, small interfering RNA; CLDN11, claudin 11; IGF2BP3, insulin-like growth factor 2 mRNA-binding protein 3; METTL3, methyltransferase like protein 3; METTL14, methyltransferase like protein 14; CRTL, control.
