# Supplementary material for: Disrupting of IGF2BP3-stabilized CLDN11 mRNA by TNF-α increases intestinal permeability in obesity-related severe acute pancreatitis
Source: Mol Med. 2025 Jan 24;31:24. doi: 10.1186/s10020-025-01078-9 (PMC11762095; doi:10.1186/s10020-025-01078-9)
Supplement: Supplementary file 2 — Supplementary Material 2 [file 10020_2025_1078_MOESM2_ESM.docx]

**
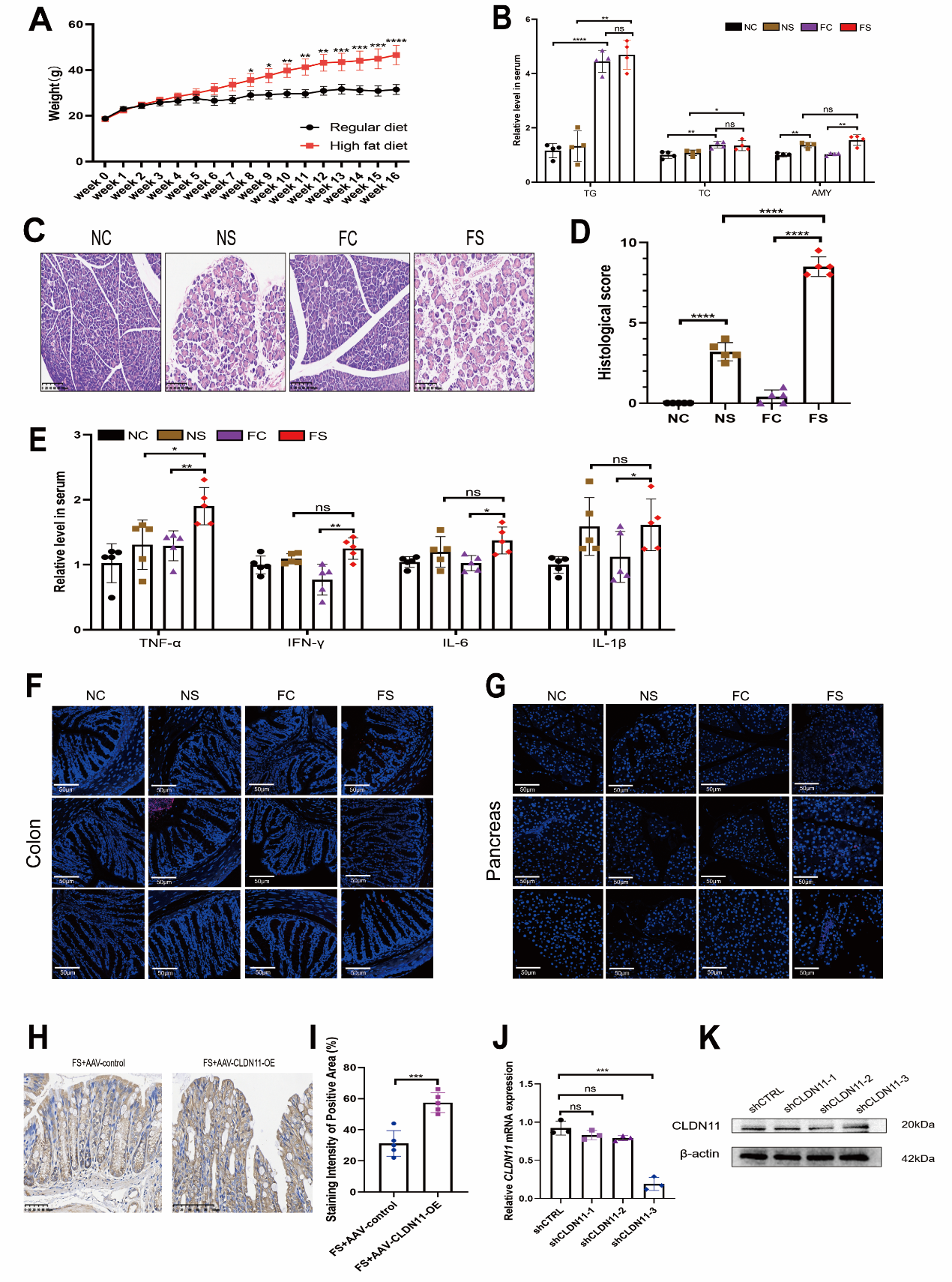
**

**Supplemental** **Fig. 1** **Obesity exacerbated SAP.** (**A**) Body weight of mice fed with high fat diet and regular diet. Each group *n*=15. (**B**) TG, TC and AMY levels in serum. Each group *n*=4. (**C**) Representative pancreatic histopathological images. (**D**) Histological scores. Each group *n*=5. (**E**) TNF-α, IFN-γ, IL-6, and IL-1β levels in serum. Each group *n*=5. (**F**) FISH images of total bacteria detected by EUB338 probe in colon in 400× magnification per field. (**G**) FISH images of total bacteria detected by EUB338 probe in pancreas in 400× magnification per field. DAPI (nucleus, blue fluorescence), EUB338 (total bacterial nucleic acid, red fluorescence). (**H**) Representative IHC images of CLDN11-stained colon sections. (**I**) Statistical analysis on the staining area of CLDN11. Each group *n*=5. (**J**) The relative mRNA level of CLDN11 in Caco-2 cells after CLDN11 knockdown. Each group *n*=3. (**K**) The relative protein level of CLDN11 in Caco-2 cells after CLDN11 knockdown. The shCLDN11-2 was used in the subsequent cellular studies. (ns, no significance, **p*<0.05, ***p*<0.01, ****p*<0.001, *****p*<0.0001)

**
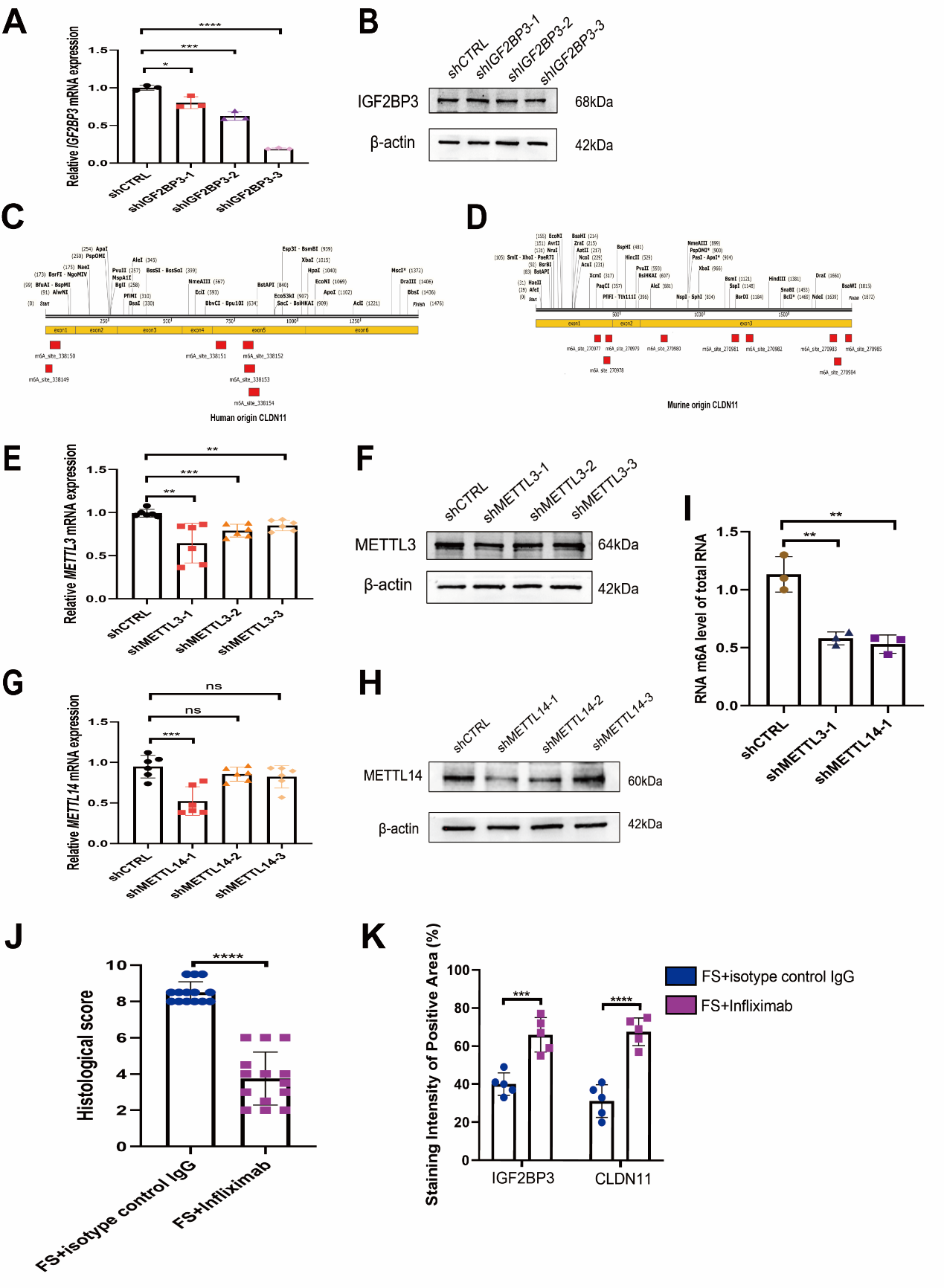
**

**Supplemental** **Fig. 2** (**A**) The relative mRNA level of IGF2BP3 in Caco-2 cells after IGF2BP3 knockdown. Each group *n*=3. (**B**) The relative protein level of IGF2BP3 in Caco-2 cells after IGF2BP3 knockdown. The shIGF2BP3-3 was used in the subsequent cellular studies. Human (**C**) and murine (**D**) origin CLDN11 mRNA have abundant RNA m6A sites. (**E**) The relative mRNA level of METTL3 in Caco-2 cells after METTL3 knockdown. Each group *n*=6. (**F**) The relative protein level of METTL3 in Caco-2 cells after METTL3 knockdown. The shMETTL3-1 was used in the subsequent cellular studies. (**G**) The relative mRNA level of METTL14 in Caco-2 cells after METTL14 knockdown. Each group *n*=6. (**H**) The relative protein level of METTL14 in Caco-2 cells after METTL14 knockdown. The shMETTL14-1 was used in the subsequent cellular studies. (**I**) RNA m6A level of total RNA in vector, METTL3 and METTL14 knockdown Caco-2 stable cells. Each group *n*=3. (**J**) Histological scores of FS + isotype control IgG and FS + Infliximab group. Each group *n*=14. (**K**) Statistical analysis on the staining area of IGF2BP3 and CLDN11 of FS + isotype control IgG and FS + Infliximab group. Each group *n*=5. (ns, no significance, **p*<0.05, ***p*<0.01, ****p*<0.001, *****p*<0.0001)
